# Proteasome function is not impaired in healthy aging of the lung

**DOI:** 10.18632/aging.100820

**Published:** 2015-10-21

**Authors:** Anne Caniard, Korbinian Ballweg, Christina Lukas, Ali Ö. Yildirim, Oliver Eickelberg, Silke Meiners

**Affiliations:** ^1^ Comprehensive Pneumology Center (CPC), University Hospital Ludwig‐Maximilians University, Helmholtz Zentrum München, Munich, Member of the German Center for Lung Research (DZL), Germany

**Keywords:** proteostasis, proteasome, emphysema, LMP2, immunoproteasome

## Abstract

Aging is the progressive loss of cellular function which inevitably leads to death. Failure of proteostasis including the decrease in proteasome function is one hallmark of aging. In the lung, proteasome activity was shown to be impaired in age‐related diseases such as chronic obstructive pulmonary disease. However, little is known on proteasome function during healthy aging. Here, we comprehensively analyzed healthy lung aging and proteasome function in wildtype, proteasome reporter and immunoproteasome knockout mice. Wildtype mice spontaneously developed senile lung emphysema while expression and activity of proteasome complexes and turnover of ubiquitinated substrates was not grossly altered in lungs of aged mice. Immunoproteasome subunits were specifically upregulated in the aged lung and the caspase‐like proteasome activity concomitantly decreased. Aged knockout mice for the LMP2 or LMP7 immunoproteasome subunits showed no alteration in proteasome activities but exhibited typical lung aging phenotypes suggesting that immunoproteasome function is dispensable for physiological lung aging in mice.

Our results indicate that healthy aging of the lung does not involve impairment of proteasome function. Apparently, the reserve capacity of the proteostasis systems in the lung is sufficient to avoid severe proteostasis imbalance during healthy aging.

## INTRODUCTION

Aging is characterized as the progressive loss of cellular, tissue and organ function that leads to increased susceptibility to disease and eventually death. In the lung, aging is an important risk factor for several “age-related” diseases such as chronic obstructive pulmonary disease (COPD), idiopathic pulmonary fibrosis (IPF), or lung cancer [1]. Importantly, lung function also physiologically declines during healthy aging [2] due to structural alterations such as the enlargement of the alveolar space leading to a condition called “senile emphysema” [2]. These changes are not restricted to humans but can also be found in aged mice during healthy aging [3]. On the cellular level several mechanisms have been identified which contribute to loss of function in aged tissues. López-Otín et al. defined nine “hallmarks of aging” that are causally linked with the aging process and together contribute to the aging phenotype, i.e. genomic instability, telomere attrition, epigenetic alterations, loss of proteostasis, deregulated nutrient sensing, mitochondrial dysfunction, cellular senescence, altered intercellular communication and stem cell exhaustion [4]. Until now, however, it remains uncertain to which extent the different hallmarks contribute to the aging phenotype of different organs and tissues.

**Figure 1:**
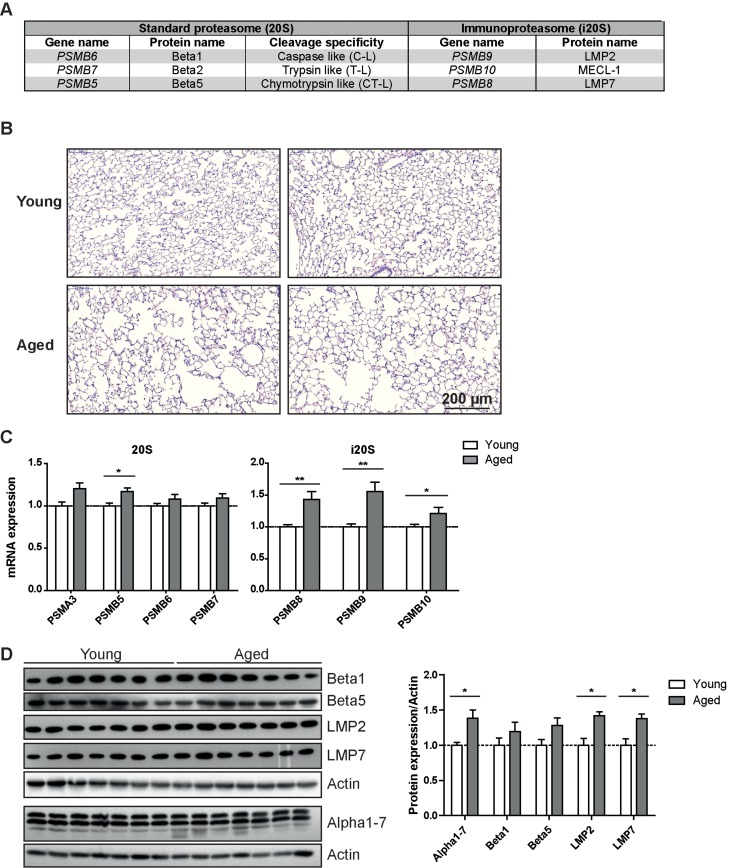
Immunoproteasome expression is increased in lung tissue of aged mice (**A**) Overview over the proteasome active site nomenclature. (**B**) Representative H&E staining of lung from young and aged wildtype C57Bl/6 mice. Images from two individual animals are depicted per group. (**C**) RTqPCR analysis of proteasome subunit mRNA expression in young and aged wildtype mice. *n* = 8+SEM. (**D**) Western blot analysis of proteasome subunit expression in young and aged wildtype mice and quantification of protein expression relative to the β − actin loading control. Bar graphs show mean+SEM.

Cellular function is intimately linked to proper protein folding and degradation of unwanted or misfolded proteins i.e. proteostasis [5]. The two main cellular protein degradation systems are the autophagy pathway and the proteasome system. The proteasome is an evolutionary conserved multi-subunit protein complex that degrades native as well as old and damaged proteins into oligopeptides and thus constitutes an integral part of protein homeostasis and protein quality control. In addition, proteasomal digestion products are used to define the cellular “self” to the immune system as they are presented as MHC class I bound antigens on the cell surface to patrolling CD8+ T-cells [6]. The proteasome core complex (also called 20S proteasome) has a cylindrical shape and consists of four heptameric rings: two outer alpha rings and two inner beta rings with the beta rings harboring the catalytic active sites β1, β2 and β5 [7, 8]. In lymphoid tissue and under specific conditions such as virus infection or IFN-γ stimulation alternative catalytic subunits are induced and incorporated into the proteasome core complex. These immunosubunits LMP2 (β1i), MECL1 (β2i), and LMP7 (β5i) replace the standard active sites and assemble into an alternative form of the proteasome known as the immunoproteasome (i20S) [9]. An overview on the catalytic active sites and their respective immuno-counterparts is given in Figure 1A. Immunoproteasomes have altered cleavage kinetic and possibly specificity [9]. They have been shown to generate peptides that have higher affinity to MHC class I molecules and thus contribute to an improved immune response against virus-infected cells [6, 9]. Furthermore immunoproteasomes have been proposed to play a protective role in the cellular response to oxidative stress, which is, however, still a matter of debate [10–13]. Beyond regulation of proteolytic activities by alternative incorporation of catalytic subunits, protein degradation by the proteasome is regulated by binding of proteasomal regulators to the catalytic 20S core particle. In particular, binding of the 19S regulator is required for the ATP-dependent degradation of polyubiquitinated proteins [7, 8]. Binding of the 19S regulator particle can occur either at one end of the 20S core or at both ends, resulting in 26S and 30S proteasome formation, respectively [8]. During aging, proteasome activity declines in several tissues such as in brain, liver, muscle, lymphocytes, and heart [14, 15]. Furthermore, overexpression of proteasome subunits in yeast and *C. elegans* was shown to increase lifespan, especially under mild stress conditions [16, 17] while flies and mice with genetically decreased proteasome activity show a premature aging phenotype [18, 19]. In addition, a very recent study reported on the particular correlation of immunoproteasome expression with maximum lifespan: 20S proteasome activity and immunoproteasome expression were found to be elevated in long-lived primate species and in rodent models with experimentally increased lifespan [20]. Surprisingly very little is known on proteasome function in the aging lung. This is even more surprising in light of the well-known age-related decline in lung function due to the progressive loss of alveolar gas exchange surface which results in a characteristic aging phenotype, i.e. senile emphysema [1, 2]. In this study, we comprehensively investigated lung aging and proteasome activity in healthy aged mice. In addition, we addressed the concept of altered immunoproteasome function in aging by analyzing histological changes of the lung and proteasome function in mice deficient for either of the immunoproteasome subunits LMP2 or LMP7.

## RESULTS

### Immunoproteasome expression is increased in lungs of aged mice

Healthy lung aging is accompanied by the enlargement of alveolar space and decreased lung function [2]. We confirmed development of senile emphysema in the lungs of 18 months old C57Bl/6 mice (Figure 1B). Aged mice concordantly showed decreased lung function parameters (Supplemental figure 1) when compared to two months old mice. These data thus recapitulate a characteristic lung aging phenotype in our aged mice. To investigate proteasome function in the lungs of aged mice we first analyzed mRNA and protein expression levels of selected proteasomal genes in total lung tissue of young and aged mice. To our surprise, proteasomal gene expression was rather increased than decreased: the standard catalytic subunits showed a trend towards increased mRNA and protein expression, which was, however, mostly not significant. (Figures 1C&D) Of note, immunoproteasome subunits were significantly increased on mRNA (Figure 1C) as well as on protein level (Figure 1D). Protein levels of the non-catalytic alpha subunits of the proteasome also increased significantly (Figure 1D). Elevated levels of immunoproteasomes are either due to an increased content of immunoproteasome expressing immune cells in the lung or due to increased expression of immunoproteasomes in resident lung cells. Collecting the loosely attached immune cells of the lung by bronchoalveolar lavage (BAL), however, revealed that there are no changes in total immune cell number or in cellular composition of the BAL between young and aged mice (Supplemental Figures 2A&B). Furthermore, the total number of LMP2 positive cells was not altered in immunofluorescence stained lung sections of young and aged animals (Supplemental Figures 2C&D) also indicating the absence of increased immune cell infiltration upon aging. Immunohistochemistry staining for the immunoproteasome subunit LMP2 rather showed elevated staining in resident cells in the lungs of aged mice which mostly appear to be alveolar plemental Figure 3). These data suggest that the macrophages. These cells have previously been shown increase in immunoproteasome expression is due to to be the most prominent immunoproteasome augmented immunoproteasome content in resident cells expressing cell type in the lung [21]. Some dispersed of the lung and not based on enhanced infiltration of staining was also apparent in lung epithelial cells (Sup-immune cell numbers into the aged lung.

### Ubiquitin-mediated protein degradation by the 26S proteasome is not altered in lungs of healthy aged mice

**Figure 2:**
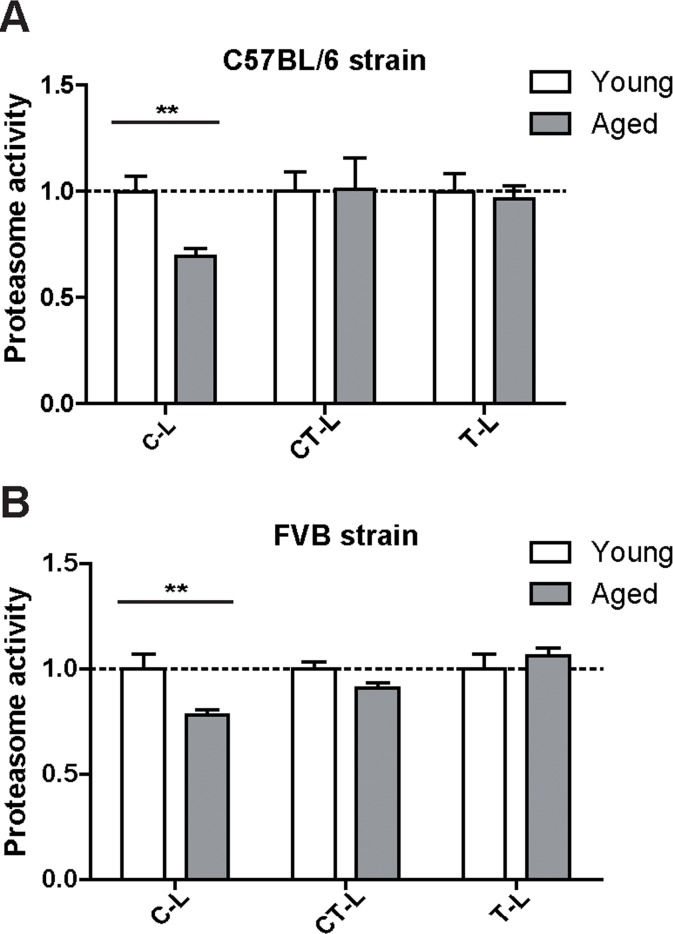
Caspase-like proteasome activity is decreased in the lungs of aged mice Cleavage of luminogenic model substrates specific for the caspase-like (C-L), chymotrypsin-like (CT-L) or trypsin-like (T-L) active site of the proteasome in lung tissue lysate of (**A**) C57Bl/6 mice *n* = 8+SEM and in (**B**) lung tissue lysate of FVB mice. *n* = 6-7+SEM.

Proteasome activity is determined by the functional assembly of all proteasome subunits into the 20S catalytic core complex and association with regulatory particles [8, 22]. To assess proteasome activity in young and aged mice we used specific peptide substrates for the different proteasome active sites. Of note and in accordance with our expression data, we did not observe any major change in the chymotrypsin- (CT-L) and trypsin-like (T-L) activities of the proteasome. Only the caspase-like (C-L) activity was slightly but significantly decreased in aged mice (Figure 2A). It is important to note that a decrease in caspase-like activity is characteristic for incorporation of the immunoproteasome subunit LMP2 into the 20S catalytic core of the proteasome [23, 24]. We confirmed this distinct activity profile of the proteasome in the aged lung in a second mouse strain, i.e. FVB mice (Figure 2B).

**Figure 3:**
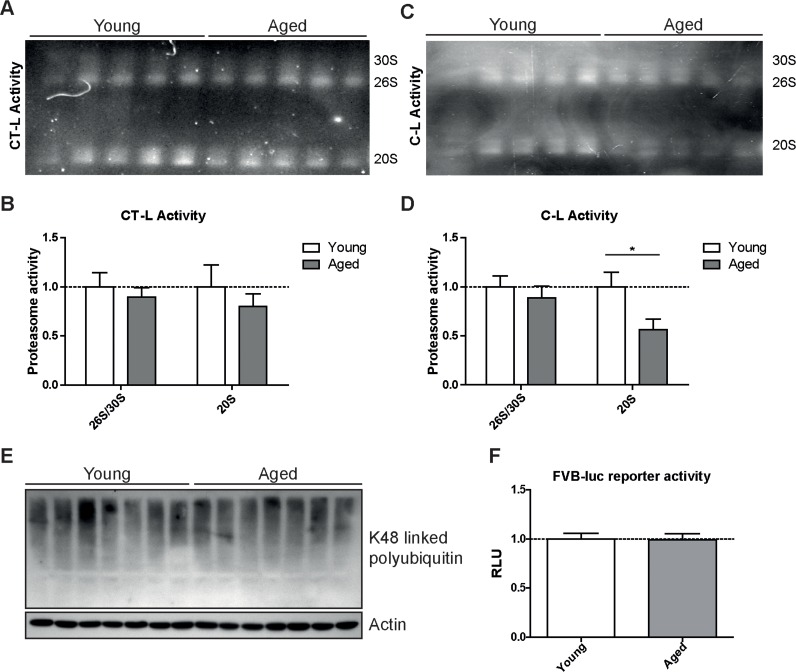
Caspase-like activity is decreased in 20S complexes but not in 26S/30S complexes of aged mice (**A**) Native gel analysis of proteasome complexes with activity overlay for the chymotrypsin-like (CT-L) proteasome activity in lung tissue lysates from young and aged C57Bl/6 mice and (**B**) quantification thereof. (**C**) Native gel analysis with activity overlay for the caspase-like (C-L) proteasome activity and (**D**) quantification thereof. The appearance of 20S double bands is possibly due to the binding of alternative proteasome regulators such as PA28 family members [57]. Bar graphs show mean+SEM. (**E**) Western blot analysis for lysin-48 (K48) linked polyubiquitinated proteins in lung tissue lysates from young and aged C57Bl/6 mice. Detection of β-actin was used as a loading control. (**F**) Luciferase activity in lung tissue of young and aged FVB-luc reporter mice. *n* = 6-7+SEM.

To confirm our unexpected findings with a second proteasome activity assay and to ascribe the catalytic activities to the distinct proteasome complexes in the lung, we performed in-gel activity assays after native electrophoretic separation of 30S, 26S and 20S proteasome complexes (Figures 3A–D). Intriguingly, the chymotrypsin-like activity of the 26S/30S and 20S proteasome complexes was very similar in young and aged lungs confirming our above results with the peptide substrates (Figure 2A). The caspase-like activity, however, was again significantly decreased predominantly in 20S complexes but not in 26S or 30S proteasomes of aged lung tissue (Figures 3C&D). In line with the observed preservation of 26S proteasome activity in lungs of aged mice, total levels of K48-polyubiquitinated proteins were not altered (Figure 3E). These results strongly indicate that ubiquitin-mediated protein degradation is not impaired in the course of healthy lung aging. We further validated these findings by using a proteasome reporter mouse to assess ubiquitin-mediated degradation of a full length and endogenously expressed proteasomal substrate in aged lung tissue. The FVB-luc mice express luciferase as a reporter gene fused to the oxygen-dependent degradation domain (ODD). Under normal conditions luciferase is degraded by the 26S proteasome resulting in a low background luminescent signal. Upon proteasome inhibition, however, luciferase accumulates leading to an increased luminescence signal [25, 26]. As we were unable to detect any change in luminescence signals, proteasomal degradation of the ODD-luc substrate is apparently not altered in the lungs of aged mice (Figure 3F).

Taken together, these results indicate that overall ubiquitin-mediated protein degradation by 26S/30S proteasome complexes in the lung is not affected in the course of healthy aging. There are, however, specific changes in the expression of catalytic subunits and in the catalytic activities of the 20S proteasomes that suggest increased assembly and activity of immunoproteasomes.

### LMP2 and LMP7 knockout mice have preserved proteasome activity but are not protected from lung aging

**Figure 4:**
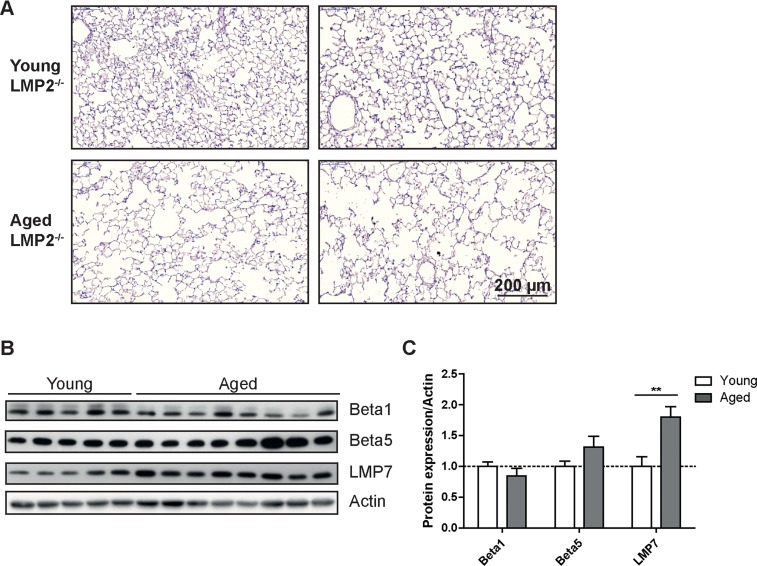
Aged LMP2 knockout mice show senile emphysema in the lung (**A**) Representative H&E staining of lungs from young and aged LMP2^−/−^ mice. Images from two individual animals are depicted per group (**B**) Western blot analysis of proteasome subunit expression in LMP2^−/−^ mice and (**C**) quantification of protein expression relative to the β − actin loading control. Bar graphs show mean+SEM.

As immunoproteasome subunits were upregulated in the aging lung (Figures 1C&D) and have been identified as important markers of the aging process [20] we investigated whether deficiency of immunoproteasome subunits affects lung aging. We used LMP2 knockout mice to analyze the relevance of elevated levels of the immunoproteasome for lung aging as in the healthy aged mice increased expression of LMP2 correlated well with a characteristic change in the caspase-like proteasome activity in the lung. Indeed, the caspase-like activity was significantly higher in young and aged LMP2 knockout mice compared to wildtype mice of the same age as shown previously [24] (Supplemental Figure 4). LMP2 deficient mice have been extensively used to analyze immunoproteasome function upon virus infection but exhibit no obvious phenotypic abnormalities at normal maintenance conditions [24]. At the age of 18 months LMP2 knockout mice showed no phenotypic signs of accelerated or decelerated aging when compared to wildtype animals (data not shown). Similarly, the lungs of aged LMP2 knockout mice developed a characteristic senile emphysema phenotype indicating that lung aging is not affected by the deficiency of this immunoproteasomal subunit (Figure 4A). In addition, we observed a very similar pattern of catalytic subunit expression in aged lungs of LMP2 deficient mice and in wildtype animals: expression of standard proteasome subunits was not altered while the remaining immunoproteasome subunits were again increased compared to young mice (Figures 4B&C).

**Figure 5:**
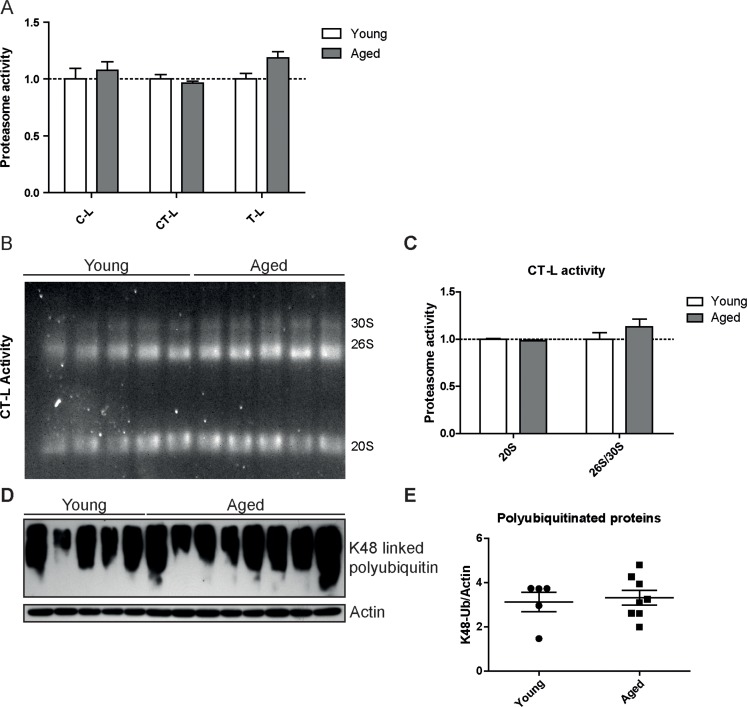
Proteasome activity is unchanged in lungs of LMP2 knockout mice (**A**) Cleavage of luminogenic model substrates specific for the caspase-like (C-L), chymotrypsin-like (CT-L) or trypsin-like (T-L) active site of the proteasome in lung tissue lysate of LMP2^−/−^ mice. *n* = 5-8+SEM. (**B**) Native gel analysis of proteasome complexes with activity overlay for CT-L activity in lung tissue lysate of LMP2^−/−^ mice and (**C**) quantification thereof. Bar graphs show mean+SEM. (**D**) Western blot analysis for lysin-48 linked polyubiquitinated proteins in lung tissue lysates from young and aged LMP2^−/−^ mice (**E**) and quantification thereof. β-Actin was used as a loading control.

Proteasome activity was not diminished in lungs of aged LMP2 knockout mice when measured in total lung lysates or in a native gel overlay assay (Figures 5A–C). In contrast to aged wildtype mice the caspase-like proteasome activity also remained unchanged (Figure 5A). Similarly, the other two proteolytic activities were not different in lungs of aged LMP2 knockout mice compared to young LMP2 knockout mice (Figures 5A&B). Accordingly, the amount of polyubiquitinated proteins was not altered in lungs of aged immunoproteasome-deficient mice compared to young animals (Figure 5D&E). Very similar to these findings in LMP2 knockout mice, aged LMP7 knockout mice showed normal organismal and lung aging and no alteration in proteasome activities (Supplemental Figure 5). In summary, lungs of aged LMP2 or LMP7 knockout mice have a very similar senile emphysema phenotype as aged wildtype mice but unchanged proteasome function. These results strongly indicate that immunoproteasome-mediated changes in proteasome activities have no functional impact on healthy aging of the lung.

## DISCUSSION

Failure of proteostasis is one of the nine hallmarks of aging. Protein homeostasis in healthy aging of the lung, however, has not been studied so far. Here, we comprehensively analyzed the function of the central maintenance system for protein homeostasis, i.e. the proteasome, in lung aging using transgenic proteasome reporter and immunoproteasome knockout mice. Importantly, overall 26S proteasome activity and ubiquitin-mediated degradation of a proteasome reporter substrate was not grossly altered in the lungs upon healthy aging. A specific decrease in only the caspase-like proteasome activity was noted and corresponded to increased expression of immuno-proteasome subunits in lungs of aged mice. Mice deficient for the immunoproteasome subunits LMP2 or LMP7 did not show any age related downregulation of proteasome activities but still displayed age-related emphysematous changes in the lung similar to wildtype mice. This argues that immunoproteasomes do not play a protective role for healthy aging of the lung.

### Proteasome function is not grossly impaired in aged lungs

Proteasome function has been shown to decrease during aging in several tissues and species [14, 15, 27–29]. Impairment of the proteasome during aging contributes to tissue damage by decreased degradation of damaged proteins thereby facilitating aggregate formation and protein toxicity [14, 15, 28, 30, 31]. Our comprehensive analysis of proteasome activity reveals that proteasome activity is not grossly impaired in lungs of healthy aged mice: while aged lungs showed prominent development of senile emphysema and age-dependent impairment of lung function, the chymotrypsin-like activities of 26S and 20S proteasome complexes, which contribute most to protein degradation, were preserved. Furthermore, ubiquitin-mediated protein degradation was not altered in aged lungs. Specifically, the proteasome reporter substrate ODD-luciferase did not accumulate upon aging in the lungs of aged reporter animals. Thus, the observed specific impairment of the caspase-like catalytic site of the proteasome in aged lungs did not affect overall ubiquitin-mediated protein degradation. Indeed, it has been reported before that the caspase-like activity has only a minor contribution of bulk protein degradation by the proteasome but rather determines degradation rates of specific substrates and the composition of the degradation products [32, 33]. A specific decrease in caspase-like activity was also consistently reported in other tissues during aging while the results for the chymotrypsin and trypsin-like activity are not consistent [27].

Preserved proteasome function in aged lungs is in contrast to results obtained for lungs of aged rats. Here, an impairment of the chymotrypsin-like activity was observed in whole lung tissue of 18–24 months old rats [14, 30]. This discrepancy might be related to substantial differences in tissue preparation and proteasome activity assays between the studies and also species-related variance. Our finding, however, accords with several reports that studied proteasome function in healthy aging specifically in the brain: Cook et al. did not detect any impairment in ubiquitin-mediated protein degradation in the brain using GFP-based proteasome reporter mice [34]. Similarly, the group of Burkard Dahlmann showed that the degradation rate of polyubiquitinated model substrates was not changed in isolated 26S proteasomes from brains of young and aged rats [35]. Furthermore, analysis of skin fibroblasts of several primate species revealed a correlation of 20S proteasome activity but not 26S activity with aging [20]. These findings, therefore, also argue in favor of a defined and tissue specific decline of proteasome function in aging.

### Immunoproteasome expression is elevated in aged lungs but does not account for the aging phenotype of the lung

We show here for the first time that immunoproteasome subunits are upregulated in lungs of aged mice. Elevated expression of immunoproteasome subunits was confirmed for mRNA and proteins levels. Increased immunoproteasome subunit expression was accompanied by increased levels of proteasome alpha subunits suggesting augmented de novo assembly of immunoptoteasomes. The increased immunoproteasome content corresponded to a reduced caspase-like activity in the aged lungs. We confirmed this finding in two different mouse strains and by using two independent assays, i.e. degradation of a luminescent and fluorescent substrate specific for caspase-like activity in tissue extracts and after separation by native gel analysis, respectively. Immunoproteasomes have been shown to exhibit a generally diminished caspase-like activity compared to standard proteasomes [23]. This specific proteolytic activity of immunoproteasomes relates to the particular function of immunoproteasomes for the generation of MHC class I epitopes: reduced caspase-like (cleavage after acidic residues) and enhanced chymotrypsin-like (cleavage after hydrophobic residues) activities of immunoproteasomes results in the preferential generation of peptides with hydrophobic C-termini. These peptides have a higher affinity to the MHC class I binding groove, resulting in improved antigen presentation and more effective clearance of virus infected cells [9]. Accordingly, caspase-like proteasome activity in the lung was restored when immunoproteasome expression is abolished in LMP2 or LMP7 knockout animals. The relative difference in caspase-like activity between wildtype and LMP2 knockout mice was particularly evident for aged mice, further arguing that the changes in caspase-like activity depend on increased immunoproteasome subunit incorporation in aging. Together, our findings implicate that in the lung the decrease in proteasome activity during aging is not based on loss of function of the proteasome but rather on altered proteasome subunit composition.

Induction of immunoproteasomes in aging has also been observed in other tissues such as muscle and brain [35, 36] and has been attributed either to enhanced recruitment of immune cells to the aged tissue or increased formation of immunoproteasomes in non-immune cells. For the lung, our immunohistochemical analysis indicated increased LMP2 expression in macrophages in the aged lung. Macrophages are the main immunoproteasome-expressing cell type in the lung [21] and in macrophages of young mice immunoproteasome expression accounts for approximately 50% of total proteasome content (I. Keller: unpublished data). Elevated abundance of macrophages in lungs of aged mice was reported before [3]. However, in this study no changes in the total number of macrophages in the BAL and no obvious difference in the number of LMP2-positive cells in the lung were found in aged lungs. These findings indicate that elevated LMP2 levels are not due to increased infiltration of immune cells in the aged lung but rather based on increased expression of immunoproteasome subunits in resident cells. This might possibly affect immune responses in the lung as altered proteasome composition affects cleavage site specificity and thus epitope processing, contributing to differential MHC class I immune surveillance. Such changes have been suggested to add to the risk of autoimmunity [37, 38]. Indeed, the prevalence of autoimmune diseases clearly rises with age [39]. Alternatively, since oxidative stress increases during aging [4, 40] and immunoproteasomes have been suggested to be involved in the degradation of oxidatively modified proteins [10–13] upregulation of immunoproteasomes may also arise from an increased need to degrade oxidatively damaged proteins. Upregulation of immunoproteasomes in non-immune cells has also been suggested recently as an evolutionary conserved feature of species longevity [20]. Its causal contribution to aging, however, has not been proven so far. We here show that both LMP2 and LMP7 knockout mice are not protected from healthy lung aging as old mice showed pronounced senile emphysema. This clearly argues that knockout of singular immunoproteasome subunits does not influence lung aging. Moreover, in lungs of aged LMP2 or LMP7 mice overall proteasome activity was restored indicating that the change in caspase-like proteasome activity and the upregulation of immunoproteasome subunits in wildtype mice during aging does not causally influence lung aging. We cannot exclude, however, that double or triple knockout mice for immunoproteasome subunits have a different phenotype. However, since the immunoproteasome pro-peptides generally favor cooperative assembly of immunoproteasomes [41, 42], knockout of a single immunoproteasome subunit also affects incorporation of other immunoproteasome catalytic sites. In contrast to our data, another study observed diminished proteasome activity in liver and brain of 12 months old LMP2 knockout mice compared to 4 months old animals. [31] In retinal epithelial cells of LMP7 knockout mice, however, proteasome activity was not altered and in LMP7 and MECL1 double knockout animals it was even elevated with age [43]. These data argue that in immunoproteasome knockout mice proteasome activity is regulated in a very tissue specific manner during aging.

### Proteostasis in healthy aging of the lung

Our data indicate that healthy aging of the lung and progressive development of senile emphysema does not involve impairment of proteasome function. Importantly, development of senile emphysema was shown to involve mechanisms that are different from disease related emphysema formation. While disease related emphysema is clearly driven by protease/antiprotease imbalance in the extracellular space [44] senile emphysema is generally described as non-destructive and is apparently not associated with elevated extracellular protease activities [2, 3, 45]. Rather intracellular factors such as a functional proteostasis network and cellular senescence have been shown to be important factors in the development of senile emphysema [3, 44]. However, since proteasome activity was not impaired during healthy lung aging the reserve proteolytic capacity of the proteasome appears to be sufficient to avoid severe proteostasis deregulation during healthy lung aging. In support of this notion, the lung structure was preserved in a transgenic mouse model with decreased chymotrypsin-like proteasome activity while these mice developed age-related phenotypes such as kyphosis, loss of skeletal muscle mass and adipose tissue and died prematurely [18, 46]. Intriguingly, recent quantitative proteomic analysis revealed that proteome composition is conserved in brain, heart and kidney upon healthy aging of mice [58]. While overall proteome composition does not significantly change upon healthy aging in mice, damaged proteins tend to aggregate during aging as recently shown in yeast and *C. elegans* [47, 48]. Such aggregates have the potential to impair proteasome function thereby leading to a vicious cycle of protein degradation and aggregate formation [47, 49, 50]. This suggests, however, that up to a specific protein damage threshold protein homeostasis may remain functional. As we did not observe any significant alteration in lung proteasome function, it is tempting to speculate that protein homeostasis remains functional upon healthy aging of the lung with little changes in proteome composition, protein aggregation and proteasomal protein degradation.

We hypothesize that dysregulation of proteostasis as a hallmark of aging is of minor importance during lung aging, at least under conditions of healthy aging. The importance of balanced proteostasis in the lung in response to environmental challenges and in age-related lung diseases, however, is well established. The aforementioned transgenic mice with decreased proteasome function showed enhanced susceptibility to cigarette smoke-induced emphysema [46]. We and others have, furthermore, reported impairment of proteasome function in response to cigarette smoke and diesel exhaust as well as in smoke and age-related chronic obstructive diseases of the lung [51–54]. Such decrease in proteolytic capacity may then tip the balance of protein homeostasis towards loss of proteostasis, further amplifying age-related and environmentally induced tissue damage.

## MATERIALS AND METHODS

### Animals and maintenance

Female C57BL/6J mice at the age of 2 or 18 months were obtained from Harlan Laboratories. LMP2^−/−^ (Psmb9^tm1Stl^ [24]) or LMP7^−/−^ (Psmb8^tm1Hjf^ [55]) mice with C57BL/6J background and ODD-luc reporter mice (129S6-Gt(ROSA)26Sor^tm1(HIF1A/luc)Kael^/J [56]) with FVB background were bought from Jackson Laboratory (Bar Harbor, ME) and housed at the Helmholtz Center Munich in rooms maintained at a constant temperature and humidity with a 12 h light cycle. Animals were allowed food and water ad libitum. All animal experiments were conducted under strict governmental and international guidelines and were approved by the local government for the administrative region of Upper Bavaria. Mouse lungs were extracted, shock-frozen in liquid nitrogen, and kept at −80°C until processing or fixed by intratracheal instillation of 4% paraformaldehyde in PBS and embedded into paraffin for hematoxylin-eosin (H&E) staining.

### Preparation of tissue lysates

To preserve proteasome activity native protein lysates were obtained by resuspension of frozen tissue in TSDG buffer (10 mM Tris/HCl, 25 mM KCl, 1.1 mM MgCl2, 0.1 mM EDTA, 1 mM DTT, 1 mM NaN3, 10% glycerol, pH 7) containing complete protease inhibitor (Roche, Basel, Switzerland). Tissue suspension was subjected to seven cycles of freezing in liquid nitrogen and thawing at room temperature. Cell debris was removed by centrifugation and protein concentration in the supernatant was assessed using the Bio-Rad Quick Start Bradford protein assay (Bio-Rad, Hercules, CA).

### Western blot analysis

For Western blot analysis, 10–20 μg of protein were subjected to electrophoresis on 10 or 12% SDS-PAGE gels and blotted onto polyvinyl-idenedifluoride (PVDF) membranes. Membranes were treated with antibodies using standard Western blot techniques. The ECL Plus Detection Reagent (GE Healthcare, Chalfont St Giles. UK) was used for chemiluminescent detection, and membranes were analyzed using X-Omat LS films (Carestream, Rochester, NY) in a Curix 60 developer (Agfa, Mortsel, Belgium). Densitometry analysis was performed using the ImageLab Software (Biorad, Hercules, CA).

Antibodies used were: anti-LMP2 (Abcam, Cambridge, UK), anti-LMP7 (Abcam), anti-beta5 (Santa Cruz Biotechnology, Dallas, TX), anti-Tbp1 (Bethyl Laboratories, Montgomery, TX), anti-PSMD7 (Abcam), anti-PSMD11 (Novus Biologicals, Littleton, CO), anti-Alpha1–7 (Abcam), HRP conjugated anti-GAPDH (Cell Signaling, Cambridge, UK), and HRP conjugated anti-β-Actin (Sigma-Aldrich, St. Louis, MO). Secondary antibodies used were HRP conjugated goat anti-mouse IgG, and HRP conjugated goat anti-rabbit IgG (GE Healthcare).

### Quantitative real-time RT-PCR

Total RNA from tissue was isolated using Roti-Quick-Kit (Carl Roth, Karlsruhe, Germany) and peqGOLD Total RNA Kit (Peqlab, Erlangen, Germany). 100–1000 ng per sample of total RNA were reverse-transcribed using random hexamers (Life Technologies) and M-MLV reverse transcriptase (Sigma-Aldrich). Quantitative PCR was performed using the SYBR Green LC480 System (Roche Diagnostics, Mannheim, Germany). The following gene-specific primers were used:

PSMA3-fw: TGAAGAAGGCTCCAATAAACGTCT
PSMA3-rv: AACGAGCATCTGCCAGCAA
PSMB5-fw: TGCTCGCTAACATGGTGTATCAGTA
PSMB5-rv: GGCCTCTCTTATCCCAGCCA
PSMB6-fw: AGACGCTGTCACTTACCAACTTGG
PSMB6-rv: AAGAGACTGGCGGCTGTGTG
PSMb7-fw: TGCCTTATGTCACCATGGGTTC
PSMB7-rv: TTCCTCCTCCATATCTGGCCTAA
PSMB8-fw: TGCTTATGCTACCCACAGAGACAA
PSMB8-rv: TTCACTTTCACCCAACCGTC
PSMB9-fw: GTACCGTGAGGACTTGTTAGCGC
PSMB9-rv: GGCTGTCGAATTAGCATCCCT
PSMB10-fw: GAAGACCGGTTCCAGCCAA
PSMB10-rv: CACTCAGGATCCCTGCTGTGAT

### Proteasome activity assay

Proteasome activity was measured with the Proteasome-Glo™ Assay System (Promega, Fitchburg, WI) according to the manufacturer's instructions. Substrates used for determination of proteasome chymotrypsin-like (CT-L), trypsin-like (T-L), and caspase-like (C-L) activities were Succinyl-leucine-leucine-valine-tyrosine-aminoluciferin (Suc-LLVY-aminoluciferin), Z-leucinearginine-arginine-aminoluciferin (Z-LRR-aminoluciferin), and Z-norleucine-proline-norleucine-aspartateaminoluciferin (Z-nLPnLD-aminoluciferin), respectively. Luminescence was measured in white flat bottom 96-well plates (BD Falcon, Franklin Lakes, NJ) in a Tristar LB 941 plate reader (Berthold Technologies, Bad Wildbad, Germany). Blank luminescence values were subtracted from each well.

### Native in-gel proteasome activity assay

Individual proteasome complexes were separated using native gel electrophoresis. 10–20 μg protein were loaded on precast 3–8% Tris-acetate gels (Life Technologies, Carlsbad, CA) and separated at 150V for 4 h at 4°C. The running buffer was Tris-borate-EDTA, containing 5 mM MgCl2, 2 mM ATP, and 1 mM DTT. For overlay activity assay gels were incubated 30 min in 50 mM Tris containing 1 mM ATP, 10 mM MgCl2, 1 mM DTT and 0.05 mM Suc-LLVY-AMC (for CT-L) or ZnLPnLD-AMC (for C-L activity).

### Statistical analysis

Two-way ANOVA with Bonferroni multiple comparison test or one-way ANOVA with Dunnett's multiple comparison test was used for statistical analysis of mRNA expression or proteasome activity. Differences in lung function were evaluated using *t*-test. Differences in protein expression data were evaluated using *t*-test or two-way ANOVA with Bonferroni multiple comparison test. All statistical analysis was performed using GraphPad Prism software (version 5.00). Significance was indicated in the figures as *: *p* < 0.05, **: *p* < 0.01 or ***: *p* < 0.001.

## SUPPLEMENTAL DATA FIGURES


