# Supplementary material for: Proteasome function is not impaired in healthy aging of the lung
Source: Aging (Albany NY). 2015 Oct 21;7(10):776–87. doi: 10.18632/aging.100820 (PMC4637206; doi:10.18632/aging.100820)
Supplement: Supplementary file 1 [file aging-07-776-s001.pdf]

SUPPLEMENTAL DATA

METHODS

Lung function analyses. Pulmonary function in mice was measured using a flexiVent system (Scireq, Montréal, Canada). Mice were anesthetized with ketamine-xylazine, tracheostomized and connected to the flexiVent system. Mice were ventilated with a tidal volume of 10 ml/kg at a frequency of 150 breaths/min in order to reach a mean lung volume similar to that of spontaneous breathing. Testing of lung mechanical properties including dynamic lung compliance and resistance was carried out by a software-generated script that took four readings per animal.

Immunohistochemistry. Immunohistochemistry for LMP2 was performed as previously described [21]. Mouse lung sections (3 µm) were deparaffinized in Xylene and rehydrated. Slides were incubated in solution containing 80% methanol and 1.8% H_2O_2 for 20 min to quench endogenous peroxidase activity. Heat-induced antigen retrieval was performed in citrate buffer (pH 6). Slides were washed with TBST buffer (20 mM Tris, 135 mM NaCl, 0.02% Tween, pH 7.6), blocked with Rodent Block M (Biocare, Concord, CA, USA) for 30 min, washed and incubated for 60 min with an LMP2 specific antibody (Abcam, Cambridge, UK). After another washing step, slides were incubated with rabbit-polymer coupled to alkaline phosphatase (Biocare) for 30 min and washed again. Vulcan Fast Red (Biocare) was used as substrate and incubated for 12 min. Hematoxylin counterstaining was performed, and slides were dehydrated and mounted in Eukitt (Sigma-Aldrich). Slides were evaluated using a MIRAX scanning system (Zeiss, Oberkochen, Germany).

Bronchoalveolar lavage cell analysis. Mouse lungs were lavaged by inserting a cannula into the trachea and instilling 4 times 1 ml of ice-cold sterile PBS into the lungs. Cells were counted and 30,000 cells were used for cytospins. These were stained according to May-Grünwald (Merck, Whitehouse Station, NJ) and cellular composition was assessed by counting approx. 100 cells per slide.

Immunofluorescence staining. Mouse lung sections (3 µm) were deparaffinized in Xylene and rehydrated and heat-induced antigen retrieval was performed in citrate buffer (pH 6). Slides were washed with PBS and lungs were permeabilized in 0.2% TritonX in PBS for 15 minutes, blocked in Roti-Immunoblock (Roth) for 1h and stained o/n with anti-LMP2 antibody (Abcam) at 4°C. Slides were then washed again, incubated with an AF-647 coupled secondary antibody (Life technologies) for 1h and with DAPI (300 nM) for 30 min and mounted using DAKO immunofluorescence mounting medium (Dako, Hamburg, Germany) and imaged using confocal laser-scanning microscopy (Zeiss LSM710, Oberkochen, Germany). Images were analyzed using the Imaris Software (Version 8.0.3, Bitplane, Zürich, Switzerland).

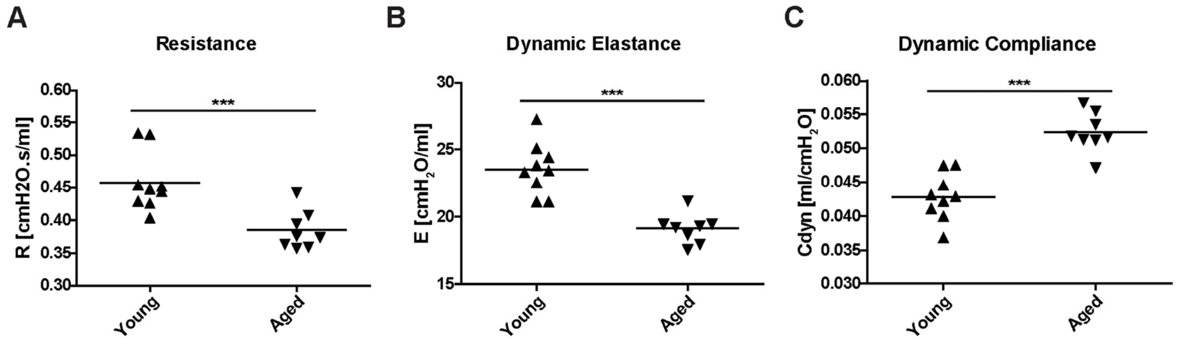

Supplemental Figure 1. Lung function in young and aged wildtype mice. Resistance (**A**), Elastance (**B**) and Compliance (**C**) of lungs of young and aged C57Bl/6 wildtype mice.

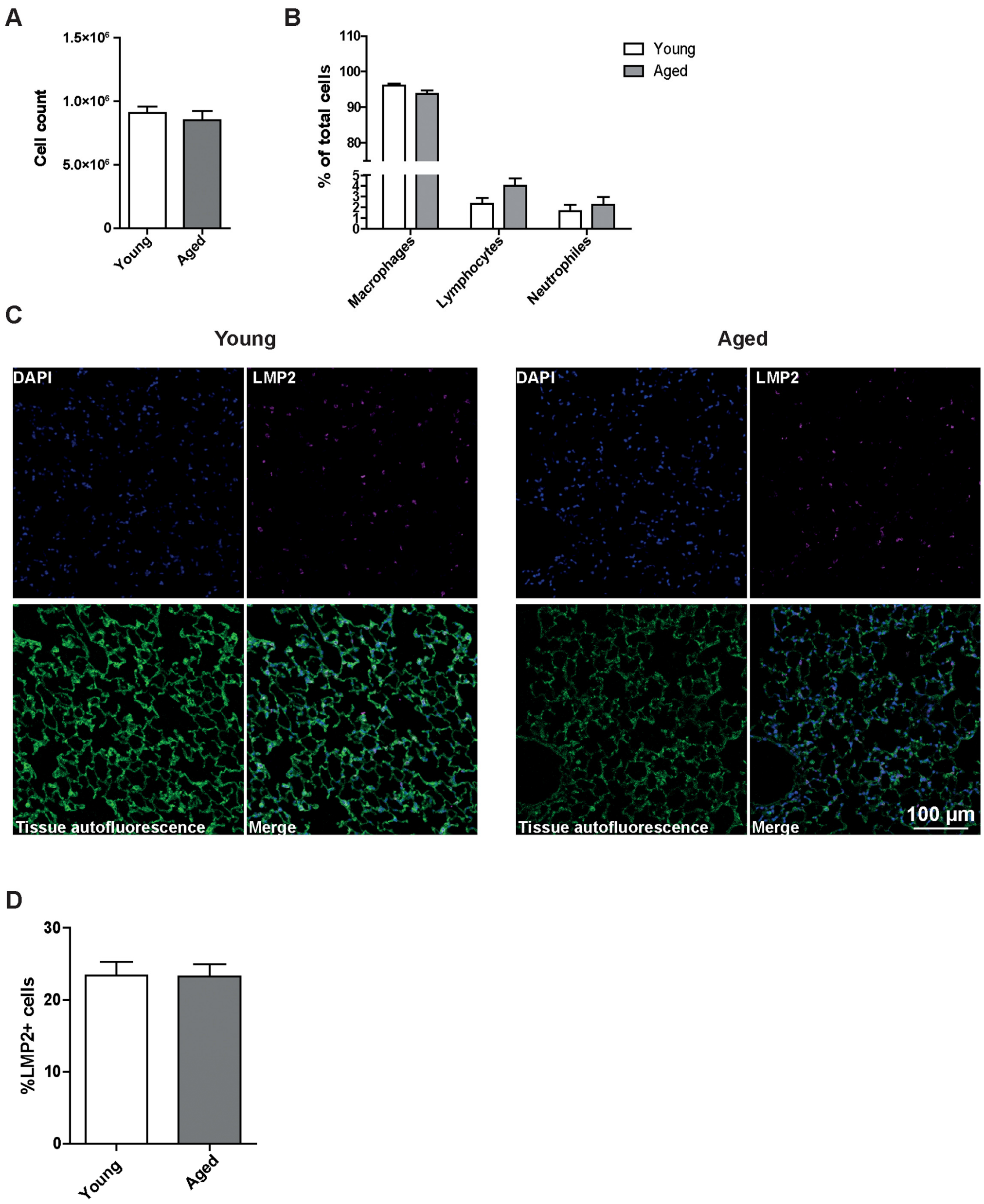

Supplemental Figure 2. No altered immune cell content in the lung of aged mice. (**A**) Total cell number in 1 ml BAL fluid from young and aged C57Bl/6 wildtype mice. (**B**) Cellular composition of BAL cells in young and aged wildtype mice. n=8-9; Bar graphs show mean+SEM. (**C**) Representative immunofluorescence images from young and aged C57Bl/6 wildtype mice. Staining show nuclei (DAPI, blue channel), LMP2 (red channel) and tissue auto-fluorescence (green channel). (**D**) Quantification of LMP2 positive cells upon immunofluorescence analysis of young and aged mice lung tissue sections using the IMARIS software. n=7-9; Bar graphs show mean+SEM.

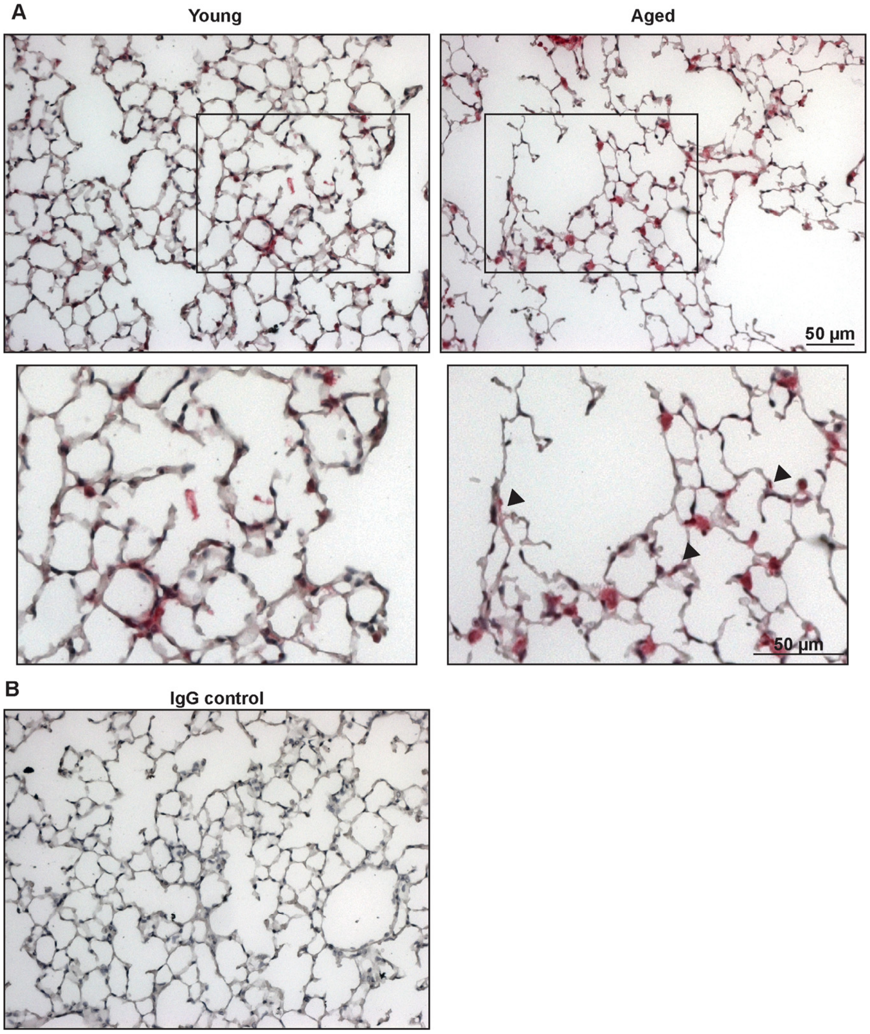

Supplemental Figure 3. LMP2 expression is mostly increased in macrophages in the aged lung. (**A**) Representative immunohistochemistry staining of lung sections of young and aged C57Bl/6 wildtype mice. LMP2 staining was mostly detected in macrophages with increased intensity in lungs of aged mice. However, in aged mice some positive staining of some epithelial structures was also observed (Arrowheads). (**B**) Immunohistochemistry control staining using an IgG control antibody.

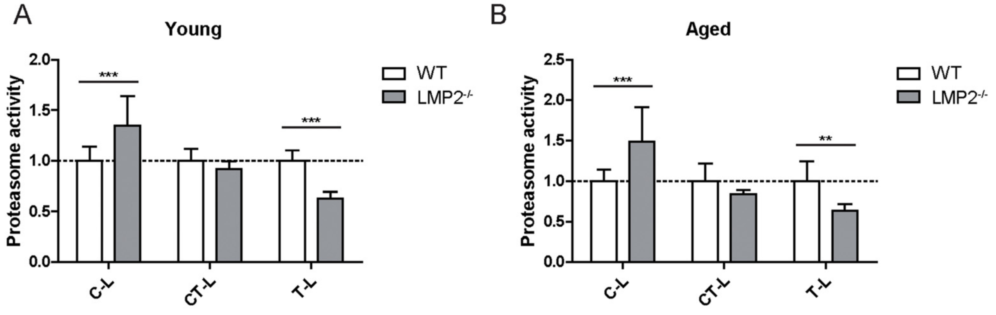

Supplemental Figure 4. Differential proteasome activity in wildtype and LMP2 knockout mice. Cleavage of luminogenic model substrates specific for the C-L, CT-L or T-L active sites of the proteasome using native lung tissue lysates of (**A**) young and (**B**) aged wildtype and LMP2 knockout mice. n=5-8+SEM**.**

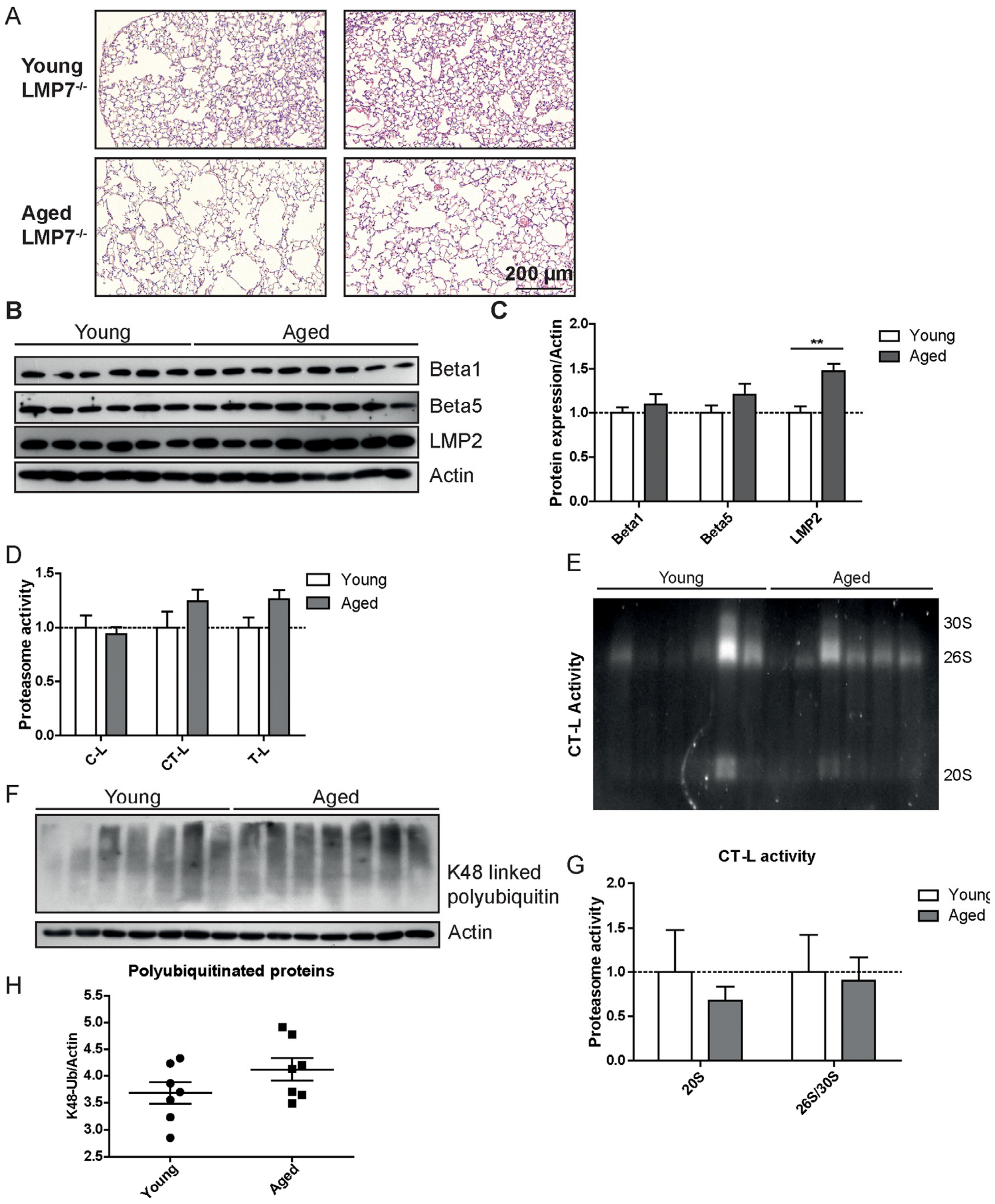

Supplemental Figure 5. LMP7 knockout mice show senile emphysema without alterations in proteasome activity. (**A**) Representative H&E staining of lungs from young and aged LMP7$^{-/-}$ mice. (**B**) Western blot analysis of proteasome subunit expression in LMP7$^{-/-}$ mice and (**C**) quantification of protein expression relative to the β-actin loading control. Bar graphs show mean+SEM. (**D**) Cleavage of luminogenic model substrates specific for the C-L, CT-L or T-L active sites of the proteasome in native lung tissue lysate of LMP7$^{-/-}$ mice. n=6-11+SEM. (**E**) Native gel analysis of proteasome complexes with activity overlay for CT-L activity in lung tissue lysate of LMP7$^{-/-}$ mice and (**G**) quantification thereof. Bar graphs show mean+SEM. (**F**) Western blot analysis for K48 linked polyubiquitinated proteins in lung tissue lysates from young and aged LMP7$^{-/-}$ mice and **(H)** quantification thereof. β-actin was used as a loading control.
